# Evaluation of the BD Max StaphSR Assay for Detecting Methicillin-Resistant Staphylococcus aureus (MRSA) and Methicillin-Susceptible S. aureus (MSSA) in ESwab-Collected Wound Samples

**DOI:** 10.1128/JCM.00641-17

**Published:** 2017-08-23

**Authors:** Suzane Silbert, Alicia Gostnell, Carly Kubasek, Raymond Widen

**Affiliations:** Esoteric Testing/R&D Laboratory, Pathology Department, Tampa General Hospital, Tampa, Florida, USA; Virginia Commonwealth University Medical Center

**Keywords:** BD Max, S. aureus, wound infections

## LETTER

Staphylococcus aureus is the most common pathogen involved in skin and soft tissue infections, and it is the principal cause of surgical site infections (1–3). Most of the laboratory methods for detecting S. aureus and methicillin-resistant S. aureus (MRSA) from wounds require incubation time and do not support rapid decisions for selection of the most appropriate procedural or therapeutic interventions (4–6). Therefore, wound infections often have negative impacts on patient outcomes— most commonly a delay or deterioration of wound healing potentially leading to sepsis (4, 7).

The BD Max StaphSR assay (BD Diagnostic Systems, Québec, Canada) performed on the BD Max system (BD Diagnostic Systems, Sparks, MD) is an FDA-cleared molecular test for detection of S. aureus DNA and MRSA DNA from nasal swab specimens collected from patients at risk of infection due to nasal colonization (8, 9). The BD Max is an automated sample-in and answer-out instrument that combines sample extraction, PCR setup, and real-time PCR on a walkaway platform. This PCR-based test can provide results in approximately 2.5 h. The objective of this study was to evaluate the BD Max StaphSR assay for the detection of S. aureus and MRSA from ESwab (Copan Diagnostics, Murrieta, CA)-collected wound samples and to compare the results to culture, our standard of care procedure. ESwab-collected wound samples are not FDA cleared for use with the BD Max StaphSR assay.

A total of 250 ESwab-collected wound samples were included in this study. All samples were tested by two different protocols: the standard of care traditional culture and the BD Max StaphSR assay on the BD Max system. For the standard of care culture, all ESwab-collected wound samples were inoculated onto BBL Trypticase soy agar with 5% sheep blood (blood agar), MacConkey II agar, chocolate II agar, Columbia CNA (colistin-nalidixic acid) agar with 5% sheep blood, and thioglycolate (THIO) broth (BD Diagnostic Systems, Sparks, MD). Swabs were rolled on the first quadrant of each medium, and plates were streaked for isolation using the 4-quadrant technique. Swabs were then wrung out in THIO. Culture plates and THIO were incubated at 35°C and observed for growth at 24 and 48 h. Bacterial colonies were identified by matrix-assisted laser desorption ionization–time of flight (MALDI-TOF) mass spectrometry, the Vitek2 GP identification card, and/or the Pastorex Staph-Plus latex agglutination test (Bio-Rad, Hercules, CA).

The BD Max StaphSR assay protocol entailed transferring an aliquot of 200 μl from the transport medium of the residual ESwab-collected sample into a BD Max sample buffer tube (SBT). SBTs were vortexed on a multiposition vortex mixer for 60 s and placed in the BD Max instrument rack along with the BD Max StaphSR assay reagent strip and reagent tubes. The entire assembly was placed in the BD Max instrument with the BD Max PCR cartridges, and the runs were initiated. Additionally, 100 μl of each ESwab sample was transferred into BBL Trypticase soy broth (TSB) with 6.5% NaCl (BD Diagnostic Systems) and incubated at 35°C for 24 h. TSB samples were saved at refrigerated temperature until culture, and PCR results were analyzed and compared. TSB samples from samples with discrepant results were plated onto both BBL CHROMagar S. aureus and BBL CHROMagar MRSA II (BD Diagnostic Systems, Sparks, MD). Colony growth was observed after 24 h of incubation at 35°C (10). Discrepant samples were also tested by an in-house-validated *mecA* and *femA* PCR, using previously published primers or probes (11) and by the Xpert MRSA/S. aureus skin and soft tissue infection (SSTI) assay on the GeneXpert system (Cepheid, Sunnyvale, CA), following the manufacturer's recommendations (1, 12, 13).

Of 250 ESwab-collected wound samples tested, 159 were negative and 83 were positive for S. aureus (Table 1) and 194 were negative and 54 were positive for MRSA (Table 2) by both tests. All 54 MRSA-positive samples were also positive for S. aureus. A total of 9 samples with discrepant results between standard of care culture and the BD Max StaphSR assay were identified. Among them, eight were negative by culture and positive by the BD Max StaphSR assay for S. aureus (Table 1). Of these culture-negative samples, one was also positive by the BD Max StaphSR assay for MRSA. Another discordant sample was positive for MRSA by culture and negative by the BD MAX StaphSR assay (Table 2). Discrepant results were resolved with additional tests as described in Table 3.

**TABLE 1:** Comparison of S. aureus results from culture and the BD Max StaphSR assay

Culture result	No. of results from BD Max StaphSR	
	Positive	Negative
Positive	83	0
Negative	8	159

**TABLE 2:** Comparison of MRSA results from culture and the BD Max StaphSR assay

Culture result	No. of results from BD Max StaphSR	
	Positive	Negative
Positive	54	1
Negative	1	194

**TABLE 3:** Discrepant result analysis

Sample	Result(s) from^*a*^:					
	Standard culture	BD Max StaphSR		CHROMagar S. aureus/MRSA	*mecA*/*femA* PCR	Xpert SSR S. aureus/MRSA
		S. aureus	MRSA			
1	−	+/FP	−/TN	−/−	−/−	−/−
2	−	+/FP	−/TN	−/−	−/−	−/−
3	−	+/FP	−/TN	−/−	−/−	−/−
4	MRSA (+)	+/TP	−/FN	−/−	+/+	+/+
5	−	+/TP	−/TN	−/−	+/−	+/−
6	−	+/TP	+/TP	−/−	+/+	+/+
7	−	+/FP	−/TN	−/−	−/−	−/−
8	−	+/TP	−/TN	−/−	−/+	+/−
9	−	+/FP	−/TN	−/−	−/−	−/−

a FP, false positive; FN, false negative; TP, true positive; TN, true negative.

Skin and soft tissue infections (SSTIs) are among the most common bacterial infections managed by clinicians, S. aureus being the most common pathogen isolated from wound infection (1, 2, 5, 7). Uncomplicated SSTIs were largely managed in outpatient settings by simple incision and drainage procedures for treatment. Traditional wound management has shown to be an imprecise form of therapy for community-associated methicillin-resistant S. aureus infections. This results in increased treatment failure, recurrent infections, local or generalized spread, and other complications (3, 4, 7). Our study evaluated the BD Max StaphSR assay to detect S. aureus and MRSA in wound samples. The BD Max StaphSR assay and culture displayed an excellent overall agreement for the detection of MRSA (99.2%) and S. aureus (96.8%) from ESwab-collected wound samples. All five S. aureus false-positive samples detected by the BD Max StaphSR assay presented threshold cycle (*C_T_*) values of ≥38, which could indicate very low bacterial load being detected. Moreover, the BD Max StaphSR assay has the ability to yield faster results than culture and can potentially facilitate earlier treatment for wound infections.
